# Dr. Oscar H. Hall

**Published:** 1912-05

**Authors:** 


Dr. Oscar H. Hall of St. Paul, Minn., a graduate of the University of Buffalo, 1868, died April 2, 1912, after a lingering illness, aged 70. He was formerly President of the Minnesota State Homoeopthic Society and a member of the state examining board. He was a veteran of the Civil War.

